# Relative Incidence of Acute Adverse Events with Ferumoxytol Compared to Other Intravenous Iron Compounds: A Matched Cohort Study

**DOI:** 10.1371/journal.pone.0171098

**Published:** 2017-01-30

**Authors:** James B. Wetmore, Eric D. Weinhandl, Jincheng Zhou, David T. Gilbertson

**Affiliations:** 1 Chronic Disease Research Group, Minneapolis Medical Research Foundation, Minneapolis, Minnesota, United States of America; 2 Division of Nephrology, Hennepin County Medical Center, Minneapolis, Minnesota, United States of America; Hospital Universitario de la Princesa, SPAIN

## Abstract

Concerns persist about adverse reactions to intravenous (IV) iron. We aimed to determine the relative safety of ferumoxytol compared to other IV iron compounds. This retrospective cohort study with propensity-score matching for patients and drug doses used the Medicare 20% random sample to identify patients (1) without chronic kidney disease (non-CKD) and (2) with non-dialysis-dependent chronic kidney disease (NDD-CKD) who received a first dose of IV iron in 2010–2012. Exposures were ferumoxytol, iron sucrose, sodium ferric gluconate, or iron dextran. Outcomes were hypersensitivity symptoms, anaphylaxis, emergency department (ED) encounters, hospitalizations, and death after acute IV iron exposure. In the primary analysis for reactions on the day of or following exposure, there was no difference in hypersensitivity symptoms (hazard ratio 1.04, 95% confidence interval 0.94–1.16) or hypotension (0.83, 0.52–1.34) between 4289 non-CKD ferumoxytol users and an equal number of users of other compounds; results were similar for 7358 NDD-CKD patients and an equal number of controls. All-cause ED encounters or hospitalizations were less common in both the non-CKD (0.56, 0.45–0.70) and NDD-CKD ferumoxytol-treated patients (0.83, 0.71–0.95). Fewer than 10 deaths occurred in both the non-CKD and NDD-CKD ferumoxytol users and in matched controls; the hazard for death did not differ significantly between ferumoxytol users and controls in the non-CKD patients (2.00, 0.33–11.97) or in the NDD-CKD patients (0.25, 0.04–1.52). Multiple sensitivity analyses showed similar results. Ferumoxytol did not appear to be associated with more adverse reactions than other compounds for the treatment of iron-deficiency anemia in both non-CKD and NDD-CKD patients.

## Introduction

Iron-deficiency anemia (IDA) is a common disorder in both the general and the chronic kidney disease (CKD) populations [1]. In patients with non-dialysis-dependent CKD (NDD-CKD), prevalence of IDA increases sharply as CKD stage worsens [2–7], and in the general population, the absolute number of affected individuals worldwide is extremely high [1,8–11]. Unfortunately, oral iron can be slow to improve iron stores [1,12], may have side effects [13,14]. and poses challenges for adherence [15], while parenteral iron can cause serious adverse events such as hypotension, anaphylaxis, and death [16–28].

An early parenteral compound in the US was high-molecular-weight iron dextran (Imferon, Fisons), which was later withdrawn from the market, while low-molecular-weight iron (INFeD, Actavis Pharma) was introduced with a US Food and Drug Administration (FDA) boxed warning (colloquially, a “black box”) in the product label [29]. While subsequent work demonstrated that high-molecular-weight iron dextran formulations conferred the greater risk [18,20,30], a perception persisted that iron dextrans in general produced unacceptable rates of serious adverse events [27]. Subsequently, ferric gluconate (Ferrlecit, Sanofi-Aventis) was introduced to the US in 1999 and iron sucrose (Venofer, American Regent) in 2000. In 2009, ferumoxytol (Feraheme, AMAG Pharmaceuticals), which was designed to more rapidly correct iron deficiency, was introduced. Despite clinical trials in patients without known multidrug allergies that suggested that ferumoxytol was no less safe than iron sucrose [25] and oral iron [31,32], reporting of spontaneous adverse events prompted the FDA to attach a boxed warning to its label [33].

Considerable controversy about the relative safety of parenteral iron compounds remains [29]. While a recent major study demonstrated that ferumoxytol was no more likely to result in anaphylaxis than other intravenous (IV) formulations, death and care encounters (e.g., emergency department [ED] encounters and hospitalizations) do not appear to have been specifically studied [34]. Another study demonstrated that ferumoxytol was not associated with greater risk of all-cause, infectious, or cardiovascular mortality [35], but this study was conducted exclusively in incident hemodialysis patients. We therefore conducted a retrospective cohort study to compare the incidence of adverse events in new users of intravenously administered ferumoxytol with incidence of events in users of other agents among Medicare beneficiaries without known kidney disease and with NDD-CKD.

## Methods

### Study Cohorts

We analyzed a 20% sample of paid Medicare Parts A and B claims in 2009–2012. We searched claims for administration of ferumoxytol (Healthcare Common Procedure Coding System [HCPCS] codes Q0138 or Q0139), iron sucrose (J1756), sodium ferric gluconate (J2916), or iron dextran (J1750). We identified patients with first new administration between January 1, 2010, and November 30, 2012 (S1 Fig), after a history of no administrations in 2009; as such, patients had a minimum of 1 year free of IV iron (i.e., encompassing all of 2009). We defined the index date as the date of first administration and the entry period as the 12 months preceding the index date. We retained patients who, during the entry period, were enrolled in Parts A and B and not Part C, and had no evidence of receiving maintenance dialysis. We stratified patients by presence of CKD, identified by ≥ 1 inpatient or post-acute facility claim or ≥ 2 outpatient facility or carrier claims with qualifying diagnosis codes (S1 Appendix) during the entry period. We followed each patient from the index date to the earliest of first administration of an alternative IV iron agent, onset of end stage renal disease, death, interruption of Parts A or B coverage, or December 15, 2012.

### Data Elements

For each IV iron-treated patient, we ascertained age, race, sex, CKD stage, numbers of ED encounters and hospital admissions, comorbid conditions, and concomitant injectable medications (the latter assessed once, on the index date). CKD stage was defined according to the most severe stage documented by diagnosis codes during the entry period. ED encounters and hospital admissions were tallied during the entry period. Comorbid conditions included all conditions except renal failure in a 19-factor adaptation of the Charlson and Elixhauser disease taxonomies [36] and histories of immunologic response. Comorbid conditions were identified by ≥ 1 inpatient or post-acute facility claim or ≥ 2 outpatient facility or carrier claims with qualifying diagnosis codes (S2 Appendix) during the entry period. Injectable medications were identified from HCPCS codes (S3 Appendix) on, and only on, the index date.

During follow-up, we identified each administration of IV iron in outpatient settings. The primary window included the date of administration and the day thereafter. Secondary windows ended on the date of administration or 3 days thereafter. For each window, we identified symptoms of hypersensitivity reactions, anaphylaxis (clinically, a form of hypersensitivity reaction), hypotension, a composite of ED encounter and hospital admission due to any cause or to cardiovascular disease (CVD), ED encounter due to any cause or to CVD, hospital admission due to any cause or to CVD, and death; these are described in substantial detail in S4 Appendix. We identified only the first occurrence of each event during the event window.

### Matching

Our primary comparison was between ferumoxytol users and non-ferumoxytol users. This contrast was selected for two reasons. First, because ferumoxytol alone received a “black box” warning, we reasoned that contrasting ferumoxytol against the other agents collectively was reasonable. Second, because we anticipated event rates to be small, we elected to increase statistical power, and thereby reduce the potential for confounding, by pooling the events for the three non-ferumoxytol compounds. However, cognizant of the fact that most clinical decisions involve the selection of one agent in head-to-head fashion against another, we also compared ferumoxytol to each of the three other compounds individually as our second approach.

To balance covariates between ferumoxytol users and non-ferumoxytol users, we used propensity score matching, separately in non-CKD and NDD-CKD patients. The propensity score match included all factors listed in Table 1. Within each stratum, ferumoxytol users were compared to non-ferumoxytol users, and, subsequently, to users of each other compound individually. In each scenario, we fit a logistic regression of ferumoxytol use. The model included only main effects. Continuously measured covariates were parameterized with linear monomials. Subsequently, we tabulated the numbers of ferumoxytol users and non-ferumoxytol users. For the mechanics of matching, we set the smaller group as the referent group and the larger group as the comparator group.

**Table 1:** Matched patient characteristics by exposure group among non-chronic kidney disease and non-dialysis-dependent chronic kidney disease patients.

	Non-CKD		NDD-CKD	
	Ferumoxytol Users	Matched Controls	Ferumoxytol Users	Matched Controls
Sample size, % (*n*)	100.0 (4289)	100.0 (4289)	100.0 (7358)	100.0 (7358)
Age, years, mean (SD)	75.0 (10.9)	74.9 (10.9)	77.1 (9.8)	77.1 (9.7)
Sex, %				
Female	67.8	67.8	56.8	56.3
Male	32.2	32.2	43.2	43.7
Race, %				
White	87.9	88.4	81.1	81.5
Black	8.3	8.2	14.2	13.6
Other	3.9	3.4	4.8	4.9
CKD stage, %				
1	—	—	1.1	1.2
2	—	—	4.4	4.4
3	—	—	50.7	49.6
4	—	—	26.5	27.4
5	—	—	4.5	4.6
Unknown	—	—	12.8	12.9
Acute care during baseline period				
Hospital admissions, any	0.6 (1.1)	0.6 (1.0)	1.1 (1.5)	1.1 (1.5)
Hospital admissions, recent, %	39.3	38.5	53.1	53.9
Emergency department, any	0.7 (1.8)	0.7 (1.6)	0.8 (1.5)	0.8 (1.6)
Emergency department, recent, %	35.0	36.2	40.4	40.3
Comorbid conditions, %				
Diabetes, any	36.4	36.9	56.3	56.9
Diabetes, complicated	10.3	10.6	33.3	33.7
Hypertension	77.6	78.2	95.2	95.3
Congestive heart failure	17.0	17.3	40.3	40.0
Cardiac arrhythmia	26.6	26.6	40.5	40.4
Peripheral vascular disease	16.3	16.5	28.7	28.4
Chronic pulmonary disease	29.8	29.4	34.4	34.2
Pulmonary vascular disease	6.1	6.3	10.2	9.9
Liver disease	7.2	7.2	6.1	6.3
Coagulopathy	10.3	10.1	11.2	10.9
Cancer, any	45.7	43.2	31.6	31.4
Cancer, metastatic	11.4	10.5	5.6	5.3
Deficiency anemia	55.0	55.1	43.6	44.0
Fluid and electrolyte disorders	21.5	20.7	41.6	41.8
Dementia	2.6	2.8	3.5	4.0
Hemiplagia or paraplegia	1.0	1.0	1.3	1.4
Psychosis	2.3	2.5	2.6	3.3
Malnutrition	11.0	10.3	10.3	9.9
Alcohol abuse	1.2	1.0	1.2	1.2
Immunological response, %				
Hypersensitivity (unrelated to food)	*	*	*	*
Drug allergy	3.1	3.1	3.9	3.8
Food, insect or latex allergy	0.7	0.9	0.7	0.7
Other allergy	2.6	2.7	2.5	2.5
Asthma	14.2	14.0	12.7	12.9
Atopic dermatitis	1.1	1.1	1.3	1.3
Allergic rhinitis	11.9	11.9	10.3	10.5
Injectable medication use (same-episode), %				
Hypersensitivity prophylaxis/treatment agents				
Ethanolamines	10.1	9.4	4.4	4.4
Glucocorticosteroids	8.5	8.0	3.2	3.3
H-2 antagonists	1.6	1.3	0.6	0.5
Anti-anemia agents				
Erythropoiesis-stimulating agents	2.6	2.6	5.9	5.9
Cobalamins	2.8	2.9	1.3	1.2
Oncologic and anti-emetic agents				
Chemotherapeutic agents	6.2	5.0	2.2	2.1
5-HT3 receptor antagonists	5.0	4.5	1.8	1.9
Other agents				
Diuretics	0.4	0.5	0.3	0.3
Heparins	2.5	2.5	1.3	1.3
Opioid agonists	*	*	0.2	0.2
Other agents	2.7	2.5	1.4	1.4

CKD, chronic kidney disease; NDD, non-dialysis-dependent, SD, standard deviation.

*Denotes fewer than 10 patients contributing. Regulations by the Centers for Medicare & Medicaid Services do not permit display.

We ordered patients in the referent group by index date and arbitrarily within index date, applying a greedy matching algorithm in the specified order. For each referent group patient, we identified his or her index date *d* and propensity score logit *r*. We retained comparator group patients whose respective index dates were within 6 months of *d*, and from this subset, we selected the comparator group patient with propensity score logit equal to *s*, such that the absolute difference between *r* and *s* was minimized; however, we applied no caliper. After a comparator group patient was selected as a matched control, the patient was removed from further matching eligibility.

### Statistical Analysis

All analyses were conducted separately for non-CKD and NDD-CKD patients. We calculated absolute standardized differences (ASDs) between ferumoxytol and non-ferumoxytol users; ASDs less than 10% are unlikely to meaningfully confound risk comparisons. We conducted risk analyses for each combination of event window and outcome, separately for first doses, non-first doses (“subsequent doses”), and all doses. The displayed incidence differences were equal to arithmetic differences between incidence proportions. Finally, we estimated modeled comparisons of risk between ferumoxytol and non-ferumoxytol users. Specifically, for each event window and event, we fit a stratified Cox proportional hazard regression. Each stratum comprised two doses: the *k*th dose received by the ferumoxytol user in a matched pair and the *k*th dose received by the non-ferumoxytol user in that pair. The time-to-event feature of the Cox regression permitted statistical accounting of the number of days between the date of IV iron administration and the date of the event (in the case of multi-day event windows). We used a sandwich covariance estimator to account for correlation of event occurrence among strata derived from the same matched pair. From the fitted model, we calculated the hazard ratio of the specified event (for use of ferumoxytol versus use of another agent), and the corresponding 95% confidence intervals and *P* values. We did not calculate the hazard ratio for any setting in which no events occurred in either treatment group. When fewer than 10 events occurred, counts are illustrated with an asterisk (*), since Centers for Medicare & Medicaid Services (CMS) regulations do not permit display.

All analyses were conducted in SAS, version 9.3 (Cary, North Carolina).

We applied to and received approval from the Human Subjects Research Committee of the Hennepin County Medical Center/Hennepin Healthcare System, Inc. Data were anonymized and the Committee waived the need for participant consent.

## Results

### Characteristics

**Fig 1:**
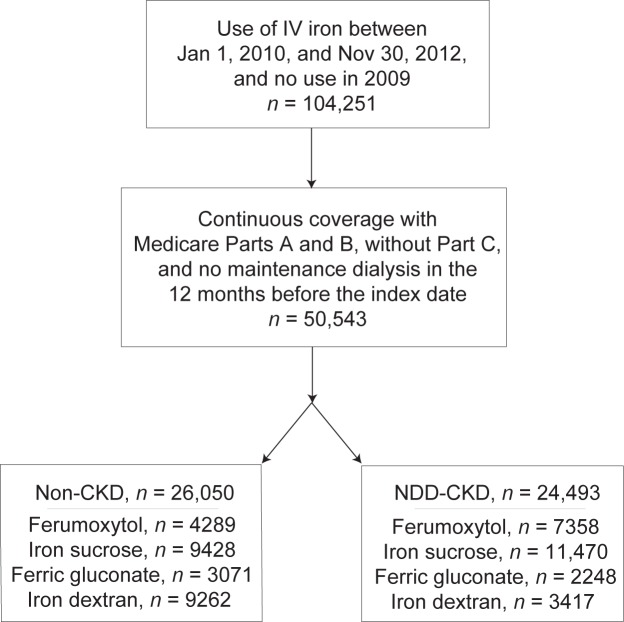
Creation of the study cohorts.

After all exclusions, 26,050 non-CKD and 24,493 NDD-CKD patients remained (Fig 1). After matching, there were 4289 non-CKD users of ferumoxytol, 9428 of iron sucrose, 3071 of ferric gluconate, and 9262 of iron dextran. Corresponding totals for the NDD-CKD patients were 7358, 11,470, 2248, and 3417. S1 Table (non-CKD cohort) and S2 Table (NDD-CKD cohort) show the characteristics of the users of each compound and the accompanying absolute standardized differences.

Characteristics of ferumoxytol users and matched users of other formulations are shown for the non-CKD and the NDD-CKD cohorts in Table 1. In the non-CKD cohort, mean age was approximately 75 years for users of ferumoxytol and of other compounds; approximately 88% of both groups were white, and about 68% were female. In the NDD-CKD cohort, mean age was approximately 77 years for both groups, about 81% were white, and approximately 56% were female. Both cohorts appeared to be well matched in the percentages seeking acute medical care in the previous year, comorbid conditions, history of immunologic response, and use of same-episode injectable medications. Additionally, in the NDD-CKD cohort, the distribution of CKD stages appeared comparable.

### Primary Analyses

**Fig 2:**
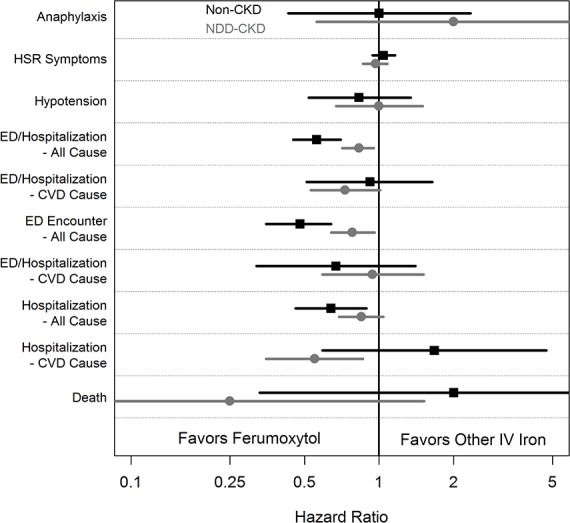
Event risk estimates, by exposure group, in the two study cohorts. CKD, chronic kidney disease; CVD, cardiovascular disease; ED, emergency department; Hosp, hospitalization; HSR, hypersensitivity reactions; IV, intravenous; NDD, non-dialysis-dependent.

Results of the primary analyses, namely the hazards for adverse events for ferumoxytol users compared to matched pooled users of other compounds on the day of or the day following administration, are shown in Tables 2 (non-CKD patients) and 3 (NDD-CKD patients). Results are shown for events occurring after a first dose and after all subsequent (“non-first”) doses, and for all doses combined (first and non-first doses). Considering all doses, among the non-CKD patients, there were no significant differences in hypersensitivity symptoms (hazard ratio [HR] 1.04, 95% confidence interval [CI] 0.94–1.16, incidence difference [ID] 0.3%), hypotension (0.83, 0.52–1.34, 0.0%), or the combined endpoint of ED encounters or hospitalizations for cardiovascular events (0.92, 0.51–1.64, 0.0%). Ferumoxytol was associated with a lower risk of all-cause hospitalizations (0.64, 0.46–0.89, -0.2%), all-cause ED encounters (0.48, 0.35–0.64, -0.5%), and the combined endpoint of all-cause ED encounters or hospitalizations (0.56, 0.45–0.70, -0.7%). For the most serious potential complications, anaphylaxis and death, fewer than 10 events of either type occurred in either the ferumoxytol-treated patients or the controls. There was no significant difference in the hazard for anaphylaxis (1.00, 0.43–2.34) or death (2.00, 0.33–11.97). The most salient results from the primary analysis, demonstrating the results for all doses, are shown graphically in Fig 2.

**Table 2:** Event risk estimates by exposure group among non-chronic-kidney-disease patients, based on outcomes the day of or the day after iron administration, derived from the Cox proportional hazards model.

	Matched Patients and Doses								
	Ferumoxytol Users			Matched Controls					
	Doses, *n*	Events, *n*	Incidence, %	Doses, *n*	Events, *n*	Incidence, %	Incidence Difference, %	HR (95% CI)	*P*
Anaphylaxis									
All doses	8405	*	*	8405	*	*	*	1.00 (0.43–2.34)	1.00
Dose: 1	4289	*	*	4289	*	*	*	3.00 (0.85–10.63)	0.089
Dose: > 1	4116	*	*	4116	*	*	*	0.33 (0.06–1.99)	0.23
HSR symptoms									
All doses	8405	548	6.5	8405	522	6.2	0.3	1.04 (0.94–1.16)	0.46
Dose: 1	4289	312	7.3	4289	318	7.4	-0.1	0.97 (0.87–1.08)	0.60
Dose: > 1	4116	236	5.7	4116	204	5.0	0.8	1.15 (0.98–1.34)	0.089
Hypotension									
All doses	8405	15	0.2	8405	18	0.2	0.0	0.83 (0.52–1.34)	0.45
Dose: 1	4289	*	*	4289	*	*	*	1.00 (0.50–2.00)	1.00
Dose: > 1	4116	*	*	4116	*	*	*	0.70 (0.36–1.34)	0.28
ED encounter or hospitalization									
All-cause									
All doses	8405	75	0.9	8405	130	1.5	-0.7	0.56 (0.45–0.70)	< 0.0001
Dose: 1	4289	41	1.0	4289	90	2.1	-1.1	0.44 (0.33–0.58)	< 0.0001
Dose: > 1	4116	34	0.8	4116	40	1.0	-0.2	0.83 (0.57–1.19)	0.30
Cardiovascular									
All doses	8405	11	0.1	8405	12	0.1	0.0	0.92 (0.51–1.64)	0.77
Dose: 1	4289	*	*	4289	*	*	*	0.63 (0.28–1.41)	0.26
Dose: > 1	4116	*	*	4116	*	*	*	1.50 (0.60–3.74)	0.38
ED encounter									
All-cause									
All doses	8405	41	0.5	8405	84	1.0	-0.5	0.48 (0.35–0.64)	< 0.0001
Dose: 1	4289	21	0.5	4289	57	1.3	-0.8	0.35 (0.24–0.52)	< 0.0001
Dose: > 1	4116	20	0.5	4116	27	0.7	-0.2	0.74 (0.46–1.19)	0.21
Cardiovascular									
All doses	8405	*	*	8405	*	*	*	0.67 (0.32–1.40)	0.29
Dose: 1	4289	*	*	4289	*	*	*	0.50 (0.18–1.40)	0.19
Dose: > 1	4116	*	*	4116	*	*	*	1.00 (0.32–3.10)	1.00
Hospitalization									
All-cause									
All doses	8405	34	0.4	8405	50	0.6	-0.2	0.64 (0.46–0.89)	0.0073
Dose: 1	4289	20	0.5	4289	36	0.8	-0.4	0.53 (0.35–0.80)	0.0023
Dose: > 1	4116	14	0.3	4116	14	0.3	0.0	0.93 (0.54–1.58)	0.79
Cardiovascular									
All doses	8405	*	*	8405	*	*	*	1.67 (0.59–4.73)	0.34
Dose: 1	4289	*	*	4289	*	*	*	1.00 (0.25–4.00)	1.00
Dose: >1	4116	*	*	4116	*	*	*	3.00 (0.50–17.95)	0.23
Death									
All doses	8405	*	*	8405	*	*	*	2.00 (0.33–11.97)	0.45
Dose: 1	4289	*	*	4289	*	*	*	2.00 (0.33–11.97)	0.45
Dose: > 1	4116	0	0.0	4116	0	0.0	0.0	—	—

CI, confidence interval; ED, emergency department; HR, hazard ratio; HSR, hypersensitivity reaction.

*Denotes fewer than 10 events contributing. Regulations by the Centers for Medicare & Medicaid Services do not permit display.

**Table 3:** Event risk estimates by exposure group among non-dialysis-dependent chronic kidney disease patients, based on outcomes the day of or the day after iron administration, derived from the Cox proportional hazards model.

	Matched Patients and Doses								
	Ferumoxytol Users			Matched Controls					
	Doses, *n*	Events, *n*	Incidence, %	Doses, *n*	Events, *n*	Incidence, %	Incidence Difference, %	HR (95% CI)	*P*
Anaphylaxis									
All doses	14,489	*	*	14,489	*	*	*	2.00 (0.56–7.09)	0.28
Dose: 1	7358	*	*	7358	*	*	*	0.50 (0.08–2.99)	0.45
Dose: > 1	7131	*	*	7131	*	*	*	—	—
HSR symptoms									
All doses	14,489	464	3.2	14,489	479	3.3	-0.1	0.97 (0.86–1.08)	0.55
Dose: 1	7358	258	3.5	7358	286	3.9	-0.4	0.90 (0.80–1.01)	0.073
Dose: > 1	7131	206	2.9	7131	193	2.7	0.2	1.07 (0.90–1.27)	0.46
Hypotension									
All doses	14,489	27	0.2	14,489	27	0.2	0.0	1.00 (0.67–1.50)	1.00
Dose: 1	7358	17	0.2	7358	16	0.2	0.0	1.06 (0.66–1.72)	0.81
Dose: > 1	7131	*	*	7131	11	0.2	*	0.91 (0.50–1.67)	0.76
ED encounter or hospitalization									
All-cause									
All doses	14,489	176	1.2	14,489	209	1.4	-0.2	0.83 (0.71–0.95)	0.0094
Dose: 1	7358	97	1.3	7358	123	1.7	-0.4	0.77 (0.64–0.93)	0.0071
Dose: > 1	7131	79	1.1	7131	86	1.2	-0.1	0.90 (0.72–1.13)	0.37
Cardiovascular									
All doses	14,489	34	0.2	14,489	45	0.3	-0.1	0.73 (0.53–1.01)	0.059
Dose: 1	7358	22	0.3	7358	24	0.3	0.0	0.92 (0.61–1.38)	0.68
Dose: > 1	7131	12	0.2	7131	21	0.3	-0.1	0.52 (0.31–0.90)	0.019
ED encounter									
All-cause									
All doses	14,489	91	0.6	14,489	116	0.8	-0.2	0.78 (0.64–0.96)	0.016
Dose: 1	7358	47	0.6	7358	65	0.9	-0.3	0.72 (0.55–0.94)	0.017
Dose: > 1	7131	44	0.6	7131	51	0.7	-0.1	0.86 (0.64–1.15)	0.31
Cardiovascular									
All doses	14,489	17	0.1	14,489	18	0.1	0.0	0.94 (0.59–1.51)	0.81
Dose: 1	7358	*	*	7358	*	*	*	1.43 (0.71–2.86)	0.31
Dose: > 1	7131	*	*	7131	11	0.2	*	0.64 (0.32–1.26)	0.20
Hospitalization									
All-cause									
All doses	14,489	87	0.6	14,489	100	0.7	-0.1	0.85 (0.69–1.04)	0.12
Dose: 1	7358	51	0.7	7358	64	0.9	-0.2	0.78 (0.60–1.02)	0.066
Dose: > 1	7131	36	0.5	7131	36	0.5	0.0	0.97 (0.70–1.35)	0.86
Cardiovascular									
All doses	14,489	17	0.1	14,489	29	0.2	-0.1	0.55 (0.35–0.86)	0.0095
Dose: 1	7358	12	0.2	7358	19	0.3	-0.1	0.63 (0.37–1.07)	0.086
Dose: >1	7131	*	*	7131	*	*	*	0.40 (0.16–0.98)	0.044
Death									
All doses	14,489	*	*	14,489	*	*	*	0.25 (0.04–1.52)	0.13
Dose: 1	7358	*	*	7358	*	*	*	—	—
Dose: > 1	7131	*	*	7131	*	*	*	0.33 (0.06–1.99)	0.23

CI, confidence interval; ED, emergency department; HR, hazard ratio; HSR, acute hypersensitivity reaction.

*Denotes fewer than 10 events contributing. Regulations by the Centers for Medicare & Medicaid Services do not permit display.

Among the NDD-CKD patients, considering all doses, there were no significant differences in hypersensitivity symptoms (0.97, 0.86–1.08, -0.1%), hypotension (1.00, 0.67–1.50, 0.0%), all-cause hospitalizations (0.85, 0.69–1.04, -0.1%), or the combined endpoint of ED encounters or hospitalizations for cardiovascular causes (0.73, 0.53–1.01, -0.1%). Ferumoxytol was associated with lower risk of all-cause ED encounters (0.78, 0.64–0.96, -0.2%) and both ED encounters or hospitalizations for all causes (0.83, 0.71–0.95, -0.2%) and cardiovascular causes (0.55, 0.35–0.86, -0.1%). Fewer than 10 deaths occurred in the ferumoxytol and the matched control groups, and fewer than 10 episodes of anaphylaxis occurred in each group. There was no significant difference in the hazards for death (0.25, 0.04–1.52) or anaphylaxis (2.00, 0.56–7.09)

### Sensitivity Analyses

Numerous sensitivity analyses were performed. In the first pair of analyses, the risk periods for an outcome were reduced and lengthened, respectively, and the results displayed in S3 and S4 Tables. For the analysis in which outcomes could occur only on the day of IV iron administration (S3 Table), there were no significant differences in the hazard of death or anaphylaxis in non-CKD or in NDD-CKD patients. In non-CKD patients, considering all doses, ferumoxytol was associated with a lower hazard for all-cause ED encounters or hospitalizations (0.52, 0.39–0.70, -0.5%) and for all-cause ED encounters (0.39, 0.27–0.57, -0.5%). In NDD-CKD patients, ferumoxytol was associated with a lower hazard of all-cause ED encounters (0.64, 0.49–0.84, -0.2%) and for the combined endpoint of all-cause ED encounters or hospitalizations (0.71, 0.59–0.87, -0.3%).

Next, the risk period was lengthened; analyses examined outcomes on the day of IV iron administration and up to 3 days later (S4 Table). Considering all doses, among non-CKD patients, there were no differences in risk of death or anaphylaxis; ferumoxytol was associated with a lower hazard for all-cause ED encounters (0.68, 0.54–0.85, -0.3%) and the combined endpoint of all-cause ED encounters or hospitalizations (0.74, 0.63–0.87, -0.4%). Among NDD-CKD patients also, ferumoxytol was associated with a lower hazard for all-cause ED encounters (0.82, 0.70–0.96, -0.2%) and the combined endpoints of all-cause ED encounters or hospitalizations (0.86, 0.77–0.97, -0.2%). Finally, ferumoxytol was compared with each of three other compounds individually (S5 Table). In no analysis was ferumoxytol associated with greater hazard for anaphylaxis or death.

## Discussion

Concerns about the safety of IV iron formulations have long persisted [25,29]. Reports of adverse events with ferumoxytol, a newer formulation available in the US, prompted the FDA to issue a boxed warning attached to the prescribing information [33,37]. To determine whether the occurrence of important adverse events differed between ferumoxytol and other commonly used compounds, we designed a large, retrospective, matched-cohort study. Overall, event rates for serious adverse events such as anaphylaxis among all compounds were very low, and we found no evidence that ferumoxytol was associated with an increased risk relative to other compounds. Results of numerous sensitivity analyses, altering the follow-up window and comparing ferumoxytol with individual alternative formulations, appear to support these findings. Thus, while our primary analysis was of ferumoxytol users in contrast to non-ferumoxytol users, the findings from the comparisons of ferumoxytol to each individual non-ferumoxytol agent were broadly concordant with those of the primary analysis.

Concern about the safety of IV iron compounds dates back to at least the early 1990s. The original high-molecular-weight iron dextran received a boxed warning in 1991 and was withdrawn from the US market, while both another high-molecular-weight iron dextran, Dexferrum, and low-molecular-weight iron dextran carried boxed warnings and required a test dose [29]. Concerns persisted with the introduction of ferumoxytol, perhaps related to the rate of IV iron administration. The iron repletion rate of ferumoxytol is relatively rapid in absolute terms; indeed, delivery of up to 30 mg of elemental iron per second was permitted according to the approved product insert until a label change in March 2015. It is possible that the relatively rapid delivery rate may initially have been a source of discomfiture among prescribers, and that ferumoxytol may therefore have been subject to heightened scrutiny regarding voluntary reporting of perceived adverse events. For example, one analysis of spontaneously reported adverse events suggested that ferumoxytol was associated with a greater risk of adverse events than non-iron-dextran-based compounds [38], but other investigators cautioned against using spontaneously reported data to make this claim [39,40].

By virtue of its design, our investigation adds important information about the relative safety of these compounds. Issues of drug safety have traditionally been explored in several types of studies, namely randomized clinical trials (RCTs), case series, analyses of adverse event reporting systems, and retrospective observational studies. While RCTs are the gold standard for demonstrating causality, they are mainly designed to test drug efficacy and typically lack the power to detect safety signals. Thus, while it is reassuring that RCTs have repeatedly demonstrated comparable safety of ferumoxytol with other compounds used for the same indication [25,41], this type of evidence alone is insufficient to demonstrate comparable safety profiles. Case series are also important sources of information, as they frequently encompass a broader range of patient types and clinical scenarios than RCTs and reflect “real-world” experiences. However, although case series generally demonstrate low risks of serious adverse events with ferumoxytol [24,42,43], the number of patients studied was likely too low to be fully reassuring.

Analysis of registry data is also a potentially important source of safety-related information. Safety registries and surveillance programs constitute adverse event reporting systems (AERS), perhaps the most well-known of which is the FDA’s MedWatch program (http://www.fda.gov/Safety/MedWatch/). AERS have been used to report on the safety of IV iron formulations [38], but this analytic approach is also not without drawbacks. First, because the AERS is fundamentally voluntary, providers may have different thresholds for initiating a report. Second, in calculating event rates using AERS data, some estimates of the number of patients treated or doses administered must be obtained outside of the AERS, since a “denominator” cannot be determined using data within the AERS. Third, voluntary reporting systems have been criticized as being subject to the “Weber effect” [44] a putative phenomenon in which newer drugs are afforded heightened scrutiny, resulting in inflated reporting of adverse events immediately following a drug’s approval. While growing awareness of the importance of reporting potential adverse events may mean that the Weber effect is less pronounced than in previous eras [45], use of the Medwatch program for judging safety has been criticized [39,40].

Large retrospective observational studies, however, can provide important insights into safety not possible using other study designs. Our study complements two recent important studies. The first found that ferumoxytol was not more likely to result in anaphylaxis than other IV iron compounds [34]. This study was conducted using the Medicare 100% sample and many years of data, permitting the generation of relatively narrow confidence intervals; our study, while substantially smaller, complements these findings by using a different matching approach, stratifying based on the presence of CKD, examining a broad range of outcomes including death and health care encounters, and including several sensitivity analyses. The decision to examine a spectrum of outcomes was deliberate; some, such as death and anaphylaxis, are characterized by high specificity but are relatively rare, while others, such as hypotension, are more common but likely much less specific. The other recent study used a facility-level approach to examine the effect of switching from either iron sucrose or ferric gluconate to ferumoxytol on outcomes in incident hemodialysis patients [35]. This analysis demonstrated no differences in all-cause, cardiovascular, or infections mortality between users of ferumoxytol and users of other IV iron compounds. Thus, three studies, each using different approaches and conducted in different patient populations, generally demonstrate broadly similar findings, strongly suggesting the ferumoxytol is not associated with a higher risk of morbidity or mortality compared to other contemporary IV iron compounds.

Our study has numerous strengths. It was conducted in two large, non-overlapping cohorts. Outcomes of interest were carefully considered: because poor specificity can be a limitation of claims data, because non-specific symptoms are often due to allergic reactions, and because physicians caring for patients with such symptoms (e.g., in the ED or the inpatient wards) may be not be fully cognizant of an exposure (IV iron) that could result in an allergic reaction, we deliberately explored a broad range of outcomes in order to overcome limitations that the authors of other studies have acknowledged [34]. Additionally, the study included a rigorous matching approach, separately considered the effects of one dose and of multiple doses, and varied the time period for outcome assessment over a range of plausible values. Finally, therapeutic alternatives to ferumoxytol were analyzed collectively and separately. Thus, the finding that ferumoxytol does not appear less safe than alternative therapies appears robust.

There are also important limitations to our approach. First, outcomes such as anaphylaxis, hypersensitivity symptoms, and hypotension may be imperfectly recorded in claims data. Additionally, we cannot be certain about the indications for subsequent ED encounters or hospitalizations, but this would not likely cause a differential bias across the drugs studied. Precise timing of medications potentially used for sensitivity prophylaxis, such as diphenhydramine, is uncertain; these medications may have been administered before or after the IV iron agent. Another limitation is that we did not have access to information on concomitant medications. We cannot rule out the possibility that adjusting for other medications, in addition to the extensive list of factors already adjusted for, might have modestly altered our results. Finally, it is uncertain whether our results can be applied to other populations.

Of note, our comparison was performed using data from 2010 to 2012, a period during which the ferumoxytol label permitted administration in as little as 17 seconds. Because the label was revised after 2012 to mandate a much slower infusion, it seems likely that, if anything, fewer adverse reactions would be observed with ferumoxytol were the study to be repeated with more contemporary data.

In conclusion, we sought to determine whether rates of a broad range of adverse events differed between users of ferumoxytol and other IV iron formulations in the outpatient setting. Overall, adverse event rates were quite low, especially for serious events such as anaphylaxis and death, and ferumoxytol did not appear to be associated with greater risk for adverse events than other compounds.

## Supporting Information

S1 Appendix International Classification of Diseases, Ninth Revision, Clinical Modification Diagnosis Codes used to identify chronic kidney disease. (PDF)

S2 Appendix International Classification of Diseases, Ninth Revision, Clinical Modification Codes used to identify comorbid conditions. (PDF)

S3 Appendix Healthcare Common Procedure Coding System codes used to identify injectable iron formulations (PDF)

S4 Appendix Definition details for anaphylaxis, symptoms of hypersensitivity reaction, hypotension, and cardiovascular disease. (PDF)

S1 Fig Study design. (DOCX)

S1 Table Characteristics of non-chronic kidney disease patients by intravenous iron agent. (DOCX)

S2 Table Characteristics of non-dialysis-dependent chronic kidney disease patients by intravenous iron agent. (DOCX)

S3 Table Event risk estimates, for ferumoxytol users versus matched controls, among non-chronic-kidney-disease and non-dialysis-dependent chronic kidney disease patients, derived from the Cox proportional hazards model: outcomes on the day of intravenous iron administration. (DOCX)

S4 Table Event risk estimates, for ferumoxytol users versus matched controls, among non-chronic-kidney-disease and non-dialysis-dependent chronic kidney disease patients, derived from the Cox proportional hazards model: outcomes on the day of intravenous iron administration and up to 3 days later. (DOCX)

S5 Table Event risk estimates, for ferumoxytol users versus individually matched users of iron sucrose, ferric gluconate, and iron dextran, among non-chronic-kidney-disease and non-dialysis-dependent chronic kidney disease patients based, derived from the Cox proportional hazards model: on outcomes the day of or the day after iron administration. (DOCX)
