# Supplementary material for: Relative Incidence of Acute Adverse Events with Ferumoxytol Compared to Other Intravenous Iron Compounds: A Matched Cohort Study
Source: PLoS One. 2017 Jan 30;12(1):e0171098. doi: 10.1371/journal.pone.0171098 (PMC5279762; doi:10.1371/journal.pone.0171098)
Supplement: S1 Appendix — (PDF) [file pone.0171098.s001.pdf]

Appendix 1. International Classification of Diseases, Ninth Revision, Clinical Modification

Diagnosis Codes used to identify chronic kidney disease

16, 16.01, 16.02, 16.03, 16.04, 16.05, 16.06
95.4
189, 189.9
223
236.91
250.4, 250.41, 250.42, 250.43
271.4
283.11
403.01, 403.11, 403.91
404.02, 404.12, 404.92, 404.03, 404.13, 404.93
440.1
442.1
447.3
572.4
580, 580.4, 580.81, 580.89, 580.9
581, 581.1, 581.2, 581.3, 581.81, 581.89, 581.9
582, 582.1, 582.2, 582.4, 582.81, 582.89, 582.9
583, 583.1, 583.2, 583.4, 583.6, 583.7, 583.81, 583.89, 583.9
584.5, 584.6, 584.7, 584.8, 584.9
585.1, 585.2, 585.3, 585.4, 585.5, 585.6, 585.9
586
587
588, 588.1, 588.81, 588.89, 588.9
591
642.1, 642.11, 642.12, 642.13, 642.14
646.2, 646.21, 646.22, 646.23, 646.24
753.12, 753.13, 753.14, 753.15, 753.16, 753.17, 753.19, 753.2, 753.21, 753.22, 753.23, 753.29
794.4
