# Supplementary material for: Relative Incidence of Acute Adverse Events with Ferumoxytol Compared to Other Intravenous Iron Compounds: A Matched Cohort Study
Source: PLoS One. 2017 Jan 30;12(1):e0171098. doi: 10.1371/journal.pone.0171098 (PMC5279762; doi:10.1371/journal.pone.0171098)
Supplement: S2 Appendix — (PDF) [file pone.0171098.s002.pdf]

Appendix 2. International Classification of Diseases, Ninth Revision, Clinical Modification

Codes used to identify comorbid conditions

Condition	Codes
History of immunologic response	995, 999.4, V13.81, 995.27, V14.0, V14.1, V14.2, V14.3, V14.4, V14.5, V14.6, V14.7, V14.8, V14.9, V15.08, 995.6, 995.61, 995.62, 995.63, 995.64, 995.65, 995.66, 995.67, 995.68, 995.69, V15.01 , V15.02 , V15.03 , V15.04, V15.05, V15.06 , V15.07 , 518.6, 708, 708.1, 708.2, 708.3, 708.4, 708.5, 708.8, 708.9, 995.3, V15.09, 477, 493, 691.8
Metastatic cancer	196, 197, 198, 199.0, 199.1
Congestive Heart Failure	398.91, 402.01, 402.11, 402.91, 404.01, 404.03, 404.11, 404.13, 404.91, 404.93, 425.4, 425.5, 425.7, 425.8, 425.9, 428
Dementia	290, 294.1, 331.2
Weight loss/malnutrition	260, 261, 262, 263, 783.2, 799.4
Hemiplegia or paraplegia	334.1, 342, 343, 344.0, 344.1, 344.2, 344.3, 344.4, 344.5, 344.6, 344.9
Alcohol abuse	291.1, 291.2, 291.3, 291.5, 291.8, 291.9, 303.0, 303.9, 305.0, 357.5, 425.5, 535.3, 571.0, 571.1, 571.2, 571.3, 980, V11.3
Any tumor	140-172, 144-195, 200-208
Cardiac arrhythmia	426.0, 426.13, 426.7, 426.9, 426.10, 426.12, 427.0, 427.1, 427.2, 427.3, 427.4, 427.6, 427.8, 427.9, 785.0, 996.01, 996.04, V45.0, V53.3
Chronic pulmonary disease	490, 491, 492, 493, 494, 495, 496, 500, 501, 502, 503, 504, 505, 416.8, 416.9, 506.4, 508.1, 508.8
Coagulopathy	286, 287.1, 287.3, 287.4, 287.5
Diabetes, any	250
Diabetes, complicated	250.4, 250.5, 250.6, 250.7, 250.8, 250.9

Deficiency anemias	280.1, 280.8, 280.9, 281
Fluid and electrolyte disorders	253.6, 276
Liver disease	070.22, 070.23, 070.32, 070.33, 070.44, 070.54, 070.6, 070.9, 570, 571, 572.2, 572.3, 572.4, 573.3, 573/4, 573.8, 573.9, V42.7
Peripheral vascular disorders	093.0, 437.3, 440, 441, 4431, 4432, 4438, 443.9, 447.1, 557.1, 557.9, V43.4
Psychosis	293.8, 295, 297, 298, 296.04, 296.14, 296.44, 296.54
Pulmonary circulation disorders	415.0, 415.1, 416, 417.0, 417.8, 417.9
HIV/AIDS	042
Hypertension	401, 402, 403, 404, 405
