# Supplementary material for: Relative Incidence of Acute Adverse Events with Ferumoxytol Compared to Other Intravenous Iron Compounds: A Matched Cohort Study
Source: PLoS One. 2017 Jan 30;12(1):e0171098. doi: 10.1371/journal.pone.0171098 (PMC5279762; doi:10.1371/journal.pone.0171098)
Supplement: S3 Appendix — (PDF) [file pone.0171098.s003.pdf]

Appendix 3. Healthcare Common Procedure Coding System codes used to identify injectable iron formulations

Code	Injectable Iron Formulation
Q0138 (non-ESRD use)	Ferumoxytol
Q0139 (ESRD use)	Ferumoxytol
J1756	Iron sucrose
J2916	Sodium ferric gluconate complex
J1750	Iron dextran

ESRD, end-stage renal disease.
