# Supplementary material for: Relative Incidence of Acute Adverse Events with Ferumoxytol Compared to Other Intravenous Iron Compounds: A Matched Cohort Study
Source: PLoS One. 2017 Jan 30;12(1):e0171098. doi: 10.1371/journal.pone.0171098 (PMC5279762; doi:10.1371/journal.pone.0171098)
Supplement: S4 Appendix — (PDF) [file pone.0171098.s004.pdf]

Appendix 4. Definition details for anaphylaxis, symptoms of hypersensitivity reaction, hypotension, and cardiovascular disease

Anaphylaxis

Anaphylaxis was identified using the FDA Mini-Sentinel Anaphylaxis Validation Workgroup algorithm (http://www.mini-sentinel.org/work_products/Validation_HealthOutcomes/Mini-Sentinel_Validation-of-Anaphylaxis-Cases.pdf)

Algorithm:

1. An emergency department or hospital admission with International Classification of Diseases, Ninth Revision, Clinical Modification (ICD-9-CM) diagnosis code 995.0 or 999.4.
2. An outpatient facility encounter with ICD-9-CM diagnosis code 995.0 or 999.4 in tandem with coding for bronchospasm (519.11), hypotension (458.9), stridor (786.1), diphenhydramine injection (Healthcare Common Procedure Coding System code J1200), epinephrine injection (J0170 or J0171), or cardiopulmonary resuscitation (ICD-9-CM procedure code 99.60 or Current Procedure Terminology code 92950).
3. An emergency department or hospital admission with ICD-9-CM diagnosis code 995.2 or 995.3 or ICD-9-CM encounter code E930-E949 in tandem with both (a) coding for bronchospasm, stridor, or diphenhydramine injection and (b) coding for hypotension, epinephrine injection, or cardiopulmonary resuscitation.

Code	Condition or Procedure
995.0	Other anaphylactic shock
999.4	Anaphylactic shock due to serum
519.11	Bronchospasm
458.9	Hypotension
786.1	Stridor

995.2	Other and unspecified adverse effect of drug, medicinal and biological substance [due] to correct medicinal substance properly administered
995.3	Allergy, unspecified
E930-E949	Drugs, medicinal and biological substances causing adverse effects in therapeutic use
99.60	Cardiopulmonary resuscitation
J1200	Diphenhydramine injection
J0170 or J0171	Epinephrine injection

Symptoms of a hypersensitivity reaction

Symptoms of a hypersensitivity reaction was identified using a component of the Nordstrom et.al. algorithm, which demonstrated 95% sensitivity and 90% specificity for hypersensitivity reactions (Nordstrom BL et. al., Pharmacoepidemiol Drug Saf. 2007;16(3):289-296):

Code	Condition
780.6	Fever
784.0	Headache
780.7	Malaise
787.0	Nausea
782.1	Rash
458.9	Hypotension

Cardiovascular disease

International Classification of Diseases, Ninth Revision, Clinical Modification Codes used for cardiovascular outcomes and cardiovascular causes of emergency department encounters and hospitalizations

401, 401.1, 401.9
402, 402.01, 402.1, 402.11, 402.9, 402.91
403, 403.01, 403.1, 403.11, 403.9, 403.91
404, 404.01, 404.02, 404.03, 404.1, 404.11, 404.12, 404.13, 404.9, 404.91, 404.92, 404.93
405.01, 405.09, 405.11, 405.19, 405.91, 405.99
410, 410.01, 410.02, 410.1, 410.11, 410.12, 410.2, 410.21, 410.22, 410.3, 410.31, 410.32, 410.4, 410.41, 410.42, 410.5, 410.51, 410.52, 410.6, 410.61, 410.62, 410.7, 410.71 , 410.72, 410.8, 410.81, 410.82, 410.9, 410.91, 410.92
411, 411.1, 411.81, 411.89
412

413, 413.1, 413.9
414, 414.01, 414.02, 414.03, 414.04, 414.05, 414.06, 414.07, 414.1, 414.11, 414.12, 414.19, 414.2, 414.3, 414.4, 414.8, 414.9
420, 420.9, 420.91, 420.99
421, 421.1, 421.9
422, 422.9, 422.91, 422.92, 422.93, 422.99
423, 423.1, 423.2, 423.3, 423.8, 423.9
424, 424.1, 424.2, 424.3, 424.9, 424.91, 424.99
425, 425.11, 425.18, 425.2, 425.3, 425.4, 425.5, 425.7, 425.8, 425.9
426, 426.1, 426.11, 426.12, 426.13, 426.2, 426.3, 426.4, 426.5, 426.51, 426.52, 426.53, 426.54, 426.6, 426.7, 426.81, 426.82, 426.89, 426.9
427, 427.1, 427.2, 427.31,427.32, 427.41, 427.42, 427.5, 427.6, 427.61, 427.69, 427.81, 427.89, 427.9
428, 428.1, 428.2, 428.21, 428.22, 428.23, 428.3, 428.31, 428.32, 428.33, 428.4, 428.41, 428.42, 428.43, 428.9
429, 429.1, 429.2, 429.3, 429.4, 429.5, 429.6, 429.71, 429.79, 429.81, 429.82, 429.83, 429.89, 429.9
785, 785.1, 785.2, 785.3, 785.4, 785.5, 785.51, 785.52, 785.59, 785.6, 785.9
786, 786.01, 786.02, 786.03, 786.04, 786.05, 786.06, 786.07, 786.09, 786.1, 786.2, 786.3, 786.31, 786.39, 786.4, 786.5, 786.51, 786.52, 786.59, 786.6, 786.7, 786.8, 786.9
