# Supplementary material for: Relative Incidence of Acute Adverse Events with Ferumoxytol Compared to Other Intravenous Iron Compounds: A Matched Cohort Study
Source: PLoS One. 2017 Jan 30;12(1):e0171098. doi: 10.1371/journal.pone.0171098 (PMC5279762; doi:10.1371/journal.pone.0171098)
Supplement: S1 Table — (DOCX) [file pone.0171098.s006.docx]

Table S1. Characteristics of non-chronic kidney disease patients by intravenous iron agent

|  | Ferumoxytol | Iron Sucrose |  | Sodium Ferric Gluconate |  | Iron Dextran |  |
| --- | --- | --- | --- | --- | --- | --- | --- |
|  | (*n* = 4289) | (*n* = 9248) |  | (*n* = 3071) |  | (*n* = 9262) |  |
| Characteristics | % or Mean ± SD | % or Mean ± SD | ASD^*^ | % or Mean± SD | ASD^†^ | % or Mean ± SD | ASD^‡^ |
| Sample size | 100.0 | 100.0 |  | 100.0 |  | 100.0 |  |
| Age, years | 75.0 ± 10.9 | 72.7 ± 12.7 | 18.7 | 72.2 ± 12.9 | 23.2 | 72.4 ± 12.0 | 22.5 |
| Sex, % |  |  |  |  |  |  |  |
| Female | 67.8 | 70.2 | 5.0 | 71.2 | 7.4 | 70.7 | 6.1 |
| Male | 32.2 | 29.8 | 5.0 | 28.8 | 7.4 | 29.3 | 6.1 |
| Race, % |  |  |  |  |  |  |  |
| White | 87.9 | 88.4 | 1.6 | 84.5 | 9.7 | 86.7 | 3.3 |
| Black | 8.3 | 7.4 | 3.5 | 11.5 | 10.6 | 9.4 | 3.8 |
| Other | 3.8 | 4.3 | 2.1 | 4.0 | 1.0 | 3.9 | 0.1 |
| Acute care during the year preceding first administration |  |  |  |  |  |  |  |
| Hospital admissions, any *n*^§^ | 0.6 ± 1.1 | 0.7 ± 1.2 | 6.9 | 0.7 ± 1.1 | 8.7 | 0.6 ± 1.1 | 0.9 |
| Hospital admission, recent % | 39.3 | 42.1 | 5.7 | 44.5 | 10.5 | 38.7 | 1.2 |
| ED encounters, any *n*^ǁ^ | 0.6 ± 1.8 | 0.8 ± 2.2 | 8.7 | 0.8 ± 2.2 | 8.8 | 0.7 ± 1.9 | 3.7 |
| ED encounter, recent % | 35.0 | 39.5 | 9.4 | 39.1 | 8.5 | 35.5 | 1.1 |
| Cardiovascular comorbidity, % |  |  |  |  |  |  |  |
| Cardiac arrhythmia | 26.6 | 27.2 | 1.3 | 26.9 | 0.7 | 25.0 | 3.7 |
| Congestive heart failure | 17.0 | 17.6 | 1.5 | 18.3 | 3.6 | 15.3 | 4.5 |
| Hypertension | 77.5 | 74.7 | 6.7 | 76.3 | 3.0 | 74.8 | 6.5 |
| Peripheral vascular disease | 16.3 | 16.8 | 1.5 | 17.1 | 2.3 | 14.8 | 4.1 |
| Pulmonary vascular disease | 6.1 | 6.3 | 0.8 | 6.7 | 2.4 | 5.5 | 2.4 |
| Hematologic and metabolic disorders, % |  |  |  |  |  |  |  |
| Coagulopathy | 10.3 | 8.1 | 7.9 | 9.6 | 2.3 | 9.8 | 1.7 |
| Deficiency anemia | 55.0 | 55.7 | 1.3 | 57.8 | 5.6 | 60.4 | 10.9 |
| Fluid and electrolyte disorders | 21.5 | 20.8 | 1.7 | 22.6 | 2.5 | 19.4 | 5.3 |
| Hepatic, pancreatic, pulmonary comorbidity, % |  |  |  |  |  |  |  |
| Chronic pulmonary disease | 29.8 | 30.9 | 2.4 | 32.4 | 5.6 | 31.2 | 3.0 |
| Diabetes (any) | 36.4 | 35.9 | 1.0 | 39.0 | 5.4 | 36.9 | 1.0 |
| Diabetes (complicated) | 10.3 | 10.5 | 0.8 | 11.2 | 3.0 | 10.1 | 0.4 |
| Liver disease | 7.2 | 8.4 | 4.3 | 8.4 | 4.3 | 7.4 | 0.6 |
| Cancer, % |  |  |  |  |  |  |  |
| Metastatic cancer | 11.4 | 8.3 | 10.4 | 9.1 | 7.5 | 8.7 | 8.7 |
| Tumor (any) | 45.7 | 31.8 | 28.8 | 34.5 | 23.0 | 35.7 | 20.4 |
| Neurologic and psychiatric comorbidity, % |  |  |  |  |  |  |  |
| Dementia | 2.6 | 2.9 | 1.9 | 2.6 | 0.1 | 2.3 | 2.1 |
| Hemiplagia or paraplegia | 1.0 | 1.5 | 4.4 | 1.2 | 2.0 | 1.1 | 0.6 |
| Psychosis | 2.3 | 3.2 | 5.6 | 3.3 | 6.3 | 2.2 | 0.7 |
| Other comorbidity, % |  |  |  |  |  |  |  |
| Alcohol abuse | 1.2 | 1.9 | 6.2 | 1.6 | 3.9 | 1.7 | 4.5 |
| Malnutrition | 11.0 | 10.5 | 1.6 | 11.0 | 0.1 | 10.3 | 2.3 |
| History of immunologic response, % |  |  |  |  |  |  |  |
| Hypersensitivity (unrelated to food) | * | * | -- | * | -- | 0.1 | -- |
| Drug allergy | 3.1 | 3.2 | 0.7 | 3.6 | 3.0 | 3.0 | 0.5 |
| Food, insect, or latex allergy | 0.7 | 0.7 | 0.1 | 0.8 | 1.4 | 0.6 | 0.9 |
| Other allergy | 2.6 | 3.0 | 2.3 | 3.7 | 6.3 | 2.8 | 1.5 |
| Allergic rhinitis | 11.9 | 12.2 | 0.9 | 11.8 | 0.1 | 13.1 | 3.6 |
| Asthma | 14.2 | 15.1 | 2.6 | 15.9 | 4.8 | 14.9 | 2.0 |
| Atopic dermatitis | 1.1 | 1.2 | 0.6 | 1.1 | 0.2 | 1.2 | 0.7 |
| Same-episode injectable medication use |  |  |  |  |  |  |  |
| Anti-anemia agents, % |  |  |  |  |  |  |  |
| Erythropoiesis-stimulating agents | 2.6 | 4.0 | 8.1 | 3.9 | 7.8 | 1.4 | 8.5 |
| Cobalamins | 2.8 | 3.4 | 3.2 | 5.3 | 12.6 | 4.9 | 10.6 |
| Oncologic and anti-emetic agents, % |  |  |  |  |  |  |  |
| Chemotherapeutic agents | 6.2 | 4.6 | 6.9 | 4.6 | 7.1 | 3.2 | 14.3 |
| 5-HT3 receptor antagonists | 5.0 | 5.5 | 2.6 | 7.1 | 8.8 | 3.8 | 5.6 |
| Hypersensitivity prophylaxis/treatment agents, % |  |  |  |  |  |  |  |
| Ethanolamines | 10.1 | 6.4 | 13.2 | 12.5 | 7.8 | 45.3 | 85.8 |
| Glucocorticosteroids | 8.5 | 6.9 | 6.1 | 12.1 | 11.9 | 34.7 | 67.1 |
| H-2 antagonists | 1.6 | 0.6 | -- | 1.3 | -- | 5.2 | -- |
| Other agents, % |  |  |  |  |  |  |  |
| Diuretics | 0.4 | 1.6 | 12.0 | 3.3 | 21.8 | 0.7 | 3.9 |
| Heparins | 2.5 | 4.3 | 9.5 | 6.5 | 19.2 | 2.8 | 1.6 |
| Opioid agonists | * | 2.4 | -- | 4.9 | -- | 0.8 | -- |
| Other agents | 2.7 | 6.5 | 18.2 | 10.7 | 32.4 | 4.3 | 8.7 |

^*^Absolute standardized difference between ferumoxytol and iron sucrose users, in percentage of 1 SD.

^†^Absolute standardized difference between ferumoxytol and sodium ferric gluconate users, in percentage of 1 SD.

^‡^Absolute standardized difference between ferumoxytol and iron dextran users, in percentage of 1 SD.

^§^Among patients with at least 1 hospital admission.

^ǁ^Among patients with at least 1 emergency room encounter.

*Denotes fewer than 10 events contributing. Regulations by the Centers for Medicare & Medicaid Services do not permit display.

ASD, absolute standardized difference; ED, emergency department; SD, standard deviation.
