# Supplementary material for: Relative Incidence of Acute Adverse Events with Ferumoxytol Compared to Other Intravenous Iron Compounds: A Matched Cohort Study
Source: PLoS One. 2017 Jan 30;12(1):e0171098. doi: 10.1371/journal.pone.0171098 (PMC5279762; doi:10.1371/journal.pone.0171098)
Supplement: S2 Table — (DOCX) [file pone.0171098.s007.docx]

Table S2. Characteristics of non-dialysis-dependent chronic kidney disease patients by intravenous iron agent

|  | Ferumoxytol | Iron Sucrose |  | Sodium Ferric Gluconate |  | Iron Dextran |  |
| --- | --- | --- | --- | --- | --- | --- | --- |
|  | (*n* = 7358) | (*n* = 11,470) |  | (*n* = 2248) |  | (*n* = 3417) |  |
| Characteristics | % or Mean ± SD | % or Mean ± SD | ASD^*^ | % or Mean ± SD | ASD^†^ | % or Mean ± SD | ASD^‡^ |
| Sample size | 100.0 | 100.0 |  | 100.0 |  | 100.0 |  |
| Age, years | 77.1 ± 9.8 | 76.4 ± 10.2 | 6.9 | 76.5 ± 10.5 | 5.8 | 76.1 ± 10.1 | 10.3 |
| Sex, % |  |  |  |  |  |  |  |
| Female | 56.8 | 57.0 | 0.4 | 56.5 | 0.5 | 57.0 | 0.5 |
| Male | 43.2 | 43.0 | 0.4 | 43.5 | 0.5 | 43.0 | 0.5 |
| Race, % |  |  |  |  |  |  |  |
| White | 81.1 | 84.2 | 8.3 | 80.6 | 1.2 | 85.4 | 11.6 |
| Black | 14.1 | 11.9 | 6.8 | 14.1 | 0.1 | 10.9 | 9.8 |
| Other | 4.8 | 3.9 | 4.2 | 5.3 | 2.4 | 3.7 | 5.4 |
| CKD stage |  |  |  |  |  |  |  |
| 1 | 1.1 | 1.1 | 0.3 | 1.3 | 1.9 | 2.4 | 9.5 |
| 2 | 4.4 | 3.6 | 4.2 | 3.9 | 2.6 | 6.0 | 7.1 |
| 3 | 50.7 | 45.9 | 9.6 | 42.7 | 16.2 | 40.0 | 21.7 |
| 4 | 26.4 | 28.1 | 3.8 | 20.8 | 13.3 | 10.9 | 40.8 |
| 5 | 4.5 | 6.1 | 7.3 | 5.6 | 5.2 | 2.9 | 8.2 |
| Unknown | 12.8 | 15.2 | 6.7 | 25.7 | 33.0 | 37.8 | 60.0 |
| Acute care during the year preceding first administration |  |  |  |  |  |  |  |
| Hospital admissions, any, *n*^§^ | 1.1 ± 1.5 | 1.2 ± 1.7 | 10.5 | 1.5 ± 2.1 | 24.8 | 1.3 ± 1.6 | 15.8 |
| Hospital admission, recent % | 53.1 | 58.3 | 10.5 | 64.1 | 22.6 | 62.3 | 18.7 |
| ED encounters, any *n*^ǁ^ | 0.8 ± 1.5 | 0.9 ± 1.9 | 7.6 | 1.0 ± 3.7 | 7.1 | 0.9 ± 2.0 | 8.2 |
| ED encounter, recent % | 40.4 | 44.1 | 7.4 | 45.7 | 10.7 | 43.9 | 7.1 |
| Cardiovascular comorbidity, % |  |  |  |  |  |  |  |
| Cardiac arrhythmia | 40.5 | 41.7 | 2.5 | 47.6 | 14.5 | 41.8 | 2.7 |
| Congestive heart failure | 40.3 | 43.5 | 6.4 | 48.2 | 15.9 | 38.7 | 3.4 |
| Hypertension | 95.1 | 95.9 | 3.6 | 95.5 | 1.5 | 92.5 | 11.0 |
| Peripheral vascular disease | 28.7 | 31.5 | 6.2 | 33.1 | 9.6 | 29.1 | 1.0 |
| Pulmonary vascular disease | 10.2 | 12.0 | 5.5 | 13.3 | 9.4 | 10.3 | 0.3 |
| Hematologic and metabolic disorders, % |  |  |  |  |  |  |  |
| Coagulopathy | 11.2 | 10.0 | 3.8 | 12.7 | 4.7 | 13.7 | 7.6 |
| Deficiency anemia | 43.6 | 41.9 | 3.5 | 52.0 | 16.8 | 58.8 | 30.8 |
| Fluid and electrolyte disorders | 41.6 | 44.5 | 5.9 | 48.1 | 13.1 | 42.7 | 2.3 |
| Hepatic, pancreatic, and pulmonary comorbidity, % |  |  |  |  |  |  |  |
| Chronic pulmonary disease | 34.4 | 37.4 | 6.2 | 41.4 | 14.5 | 39.4 | 10.3 |
| Diabetes (any) | 56.3 | 58.7 | 5.0 | 57.7 | 2.8 | 53.7 | 5.1 |
| Diabetes (complicated) | 33.3 | 35.8 | 5.3 | 34.5 | 2.6 | 28.2 | 11.0 |
| Liver disease | 6.1 | 6.5 | 1.6 | 6.8 | 2.9 | 8.1 | 7.8 |
| Cancer, % |  |  |  |  |  |  |  |
| Metastatic cancer | 5.6 | 4.4 | 5.4 | 7.2 | 6.5 | 9.5 | 14.5 |
| Tumor (any) | 31.6 | 26.2 | 12.0 | 31.5 | 0.2 | 40.7 | 19.0 |
| Neurologic and psychiatric comorbidity, % |  |  |  |  |  |  |  |
| Dementia | 3.5 | 4.2 | 3.5 | 4.8 | 6.4 | 4.5 | 5.2 |
| Hemiplagia or paraplegia | 1.3 | 1.6 | 2.8 | 1.4 | 1.1 | 1.4 | 1.1 |
| Psychosis | 2.6 | 3.6 | 5.9 | 3.8 | 6.7 | 3.1 | 3.0 |
| Other comorbidity, % |  |  |  |  |  |  |  |
| Alcohol abuse | 1.2 | 1.6 | 3.5 | 1.5 | 2.5 | 1.8 | 5.4 |
| Malnutrition | 10.2 | 9.9 | 1.0 | 12.8 | 7.9 | 13.7 | 10.7 |
| History of immunologic response, % |  |  |  |  |  |  |  |
| Hypersensitivity (unrelated to food) | * | * | -- | * | -- | * | -- |
| Drug allergy | 3.9 | 4.1 | 1.3 | 5.4 | 7.3 | 5.0 | 5.3 |
| Food, insect, or latex allergy | 0.7 | 0.7 | 0.2 | 0.9 | 2.8 | 0.6 | 1.6 |
| Other allergy | 2.5 | 2.6 | 0.5 | 3.0 | 3.4 | 2.6 | 1.0 |
| Allergic rhinitis | 10.3 | 9.1 | 4.2 | 9.8 | 1.7 | 11.8 | 4.8 |
| Asthma | 12.7 | 14.0 | 3.8 | 16.2 | 10.0 | 14.7 | 6.0 |
| Atopic dermatitis | 1.3 | 1.2 | 0.8 | 1.2 | 0.4 | 1.0 | 2.1 |
| Same-episode injectable medication use |  |  |  |  |  |  |  |
| Anti-anemia agents, % |  |  |  |  |  |  |  |
| Erythropoiesis-stimulating agents | 5.9 | 9.1 | 11.9 | 11.9 | 21.1 | 4.9 | 4.8 |
| Cobalamins | 1.3 | 1.5 | 2.0 | 1.9 | 4.7 | 4.2 | 18.2 |
| Oncologic and anti-emetic agents, % |  |  |  |  |  |  |  |
| Chemotherapeutic agents | 2.2 | 2.0 | 1.7 | 3.1 | 5.7 | 2.8 | 3.5 |
| 5-HT3 receptor antagonists | 1.8 | 2.6 | 5.2 | 5.0 | 17.6 | 3.5 | 10.3 |
| Hypersensitivity prophylaxis/treatment agents, % |  |  |  |  |  |  |  |
| Ethanolamines | 4.4 | 3.1 | 7.0 | 9.2 | 19.1 | 43.2 | 102.3 |
| Glucocorticosteroids | 3.2 | 2.7 | 2.9 | 7.4 | 18.9 | 31.3 | 80.0 |
| H-2 antagonists | 0.6 | 0.3 | 3.5 | 0.8 | 3.1 | 4.4 | 24.5 |
| Other agents, % |  |  |  |  |  |  |  |
| Diuretics | 0.3 | 1.6 | 14.3 | 5.2 | 30.9 | 1.2 | 10.8 |
| Heparins | 1.3 | 3.0 | 12.2 | 7.1 | 29.6 | 2.8 | 10.7 |
| Opioid agonists | 0.2 | 1.4 | 14.3 | 4.2 | 28.0 | 0.7 | 8.2 |
| Other agents | 1.3 | 3.9 | 16.1 | 9.9 | 37.9 | 3.7 | 14.8 |

^*^Absolute standardized difference between ferumoxytol and iron sucrose users, in percentage of 1 SD.

^†^Absolute standardized difference between ferumoxytol and sodium ferric gluconate users, in percentage of 1 SD.

^‡^Absolute standardized difference between ferumoxytol and iron dextran users, in percentage of 1 SD.

^§^Among patients with at least 1 hospital admission.

^ǁ^Among patients with at least 1 emergency room encounter.

* Denotes fewer than 10 events contributing. Regulations by the Centers for Medicare & Medicaid Services do not permit display.

ASD, absolute standardized difference; ED, emergency department; SD, standard deviation.
