# Supplementary material for: Relative Incidence of Acute Adverse Events with Ferumoxytol Compared to Other Intravenous Iron Compounds: A Matched Cohort Study
Source: PLoS One. 2017 Jan 30;12(1):e0171098. doi: 10.1371/journal.pone.0171098 (PMC5279762; doi:10.1371/journal.pone.0171098)
Supplement: S3 Table — (DOCX) [file pone.0171098.s008.docx]

Table S3. Event risk estimates, for ferumoxytol users versus matched controls, among non-chronic-kidney-disease and non-dialysis-dependent chronic kidney disease patients, derived from the Cox proportional hazards model: outcomes on the day of intravenous iron administration

|  | Non-CKD patients | | | NDD CKD Patients | | |
| --- | --- | --- | --- | --- | --- | --- |
|  | Incidence Difference, % | HR (95% CI) | *P* | Incidence Difference, % | HR (95% CI) | *P* |
| Anaphylaxis |  |  |  |  |  |  |
| All doses | * | 0.75 (0.30-1.85) | 0.53 | * | 4.00 (0.66-24.37) | 0.13 |
| Dose: 1 | * | 2.00 (0.71-5.62) | 0.19 | * | 1.00 (0.14-7.10) | 1.00 |
| Dose: > 1 | * | 0.33 (0.06-1.99) | 0.23 | * | -- | -- |
| HSR Symptoms |  |  |  |  |  |  |
| All doses | 0.4 | 1.07 (0.96-1.20) | 0.21 | -0.1 | 0.98 (0.87-1.11) | 0.78 |
| Dose: 1 | 0.1 | 1.01 (0.90-1.13) | 0.85 | -0.2 | 0.95 (0.83-1.07) | 0.38 |
| Dose: > 1 | 0.8 | 1.17 (0.99-1.38) | 0.064 | 0.1 | 1.04 (0.85-1.26) | 0.72 |
| Hypotension |  |  |  |  |  |  |
| All doses | 0.0 | 1.00 (0.58-1.72) | 1.00 | 0.0 | 0.90 (0.56-1.45) | 0.66 |
| Dose: 1 | * | 1.17 (0.54-2.53) | 0.70 | * | 0.77 (0.43-1.38) | 0.38 |
| Dose: > 1 | * | 0.83 (0.39-1.79) | 0.64 | * | 1.14 (0.56-2.35) | 0.72 |
| ED encounter or hospitalization |  |  |  |  |  |  |
| All-cause |  |  |  |  |  |  |
| All doses | -0.5 | 0.52 (0.39-0.70) | < 0.0001 | -0.3 | 0.71 (0.59-0.87) | 0.0008 |
| Dose: 1 | -0.9 | 0.34 (0.24-0.50) | < 0.0001 | -0.5 | 0.58 (0.45-0.75) | < 0.0001 |
| Dose: > 1 | 0.0 | 1.00 (0.61-1.63) | 1.00 | 0.0 | 0.96 (0.70-1.30) | 0.77 |
| Cardiovascular |  |  |  |  |  |  |
| All doses | * | 0.63 (0.28-1.41) | 0.26 | 0.0 | 0.92 (0.61-1.38) | 0.68 |
| Dose: 1 | * | 0.75 (0.26-2.18) | 0.60 | 0.0 | 0.80 (0.47-1.37) | 0.42 |
| Dose: > 1 | * | 0.50 (0.14-1.77) | 0.28 | * | 1.11 (0.59-2.10) | 0.75 |
| ED encounter |  |  |  |  |  |  |
| All-cause |  |  |  |  |  |  |
| All doses | -0.5 | 0.39 (0.27-0.57) | < 0.0001 | -0.2 | 0.64 (0.49-0.84) | 0.0012 |
| Dose: 1 | -0.8 | 0.26 (0.15-0.42) | < 0.0001 | -0.3 | 0.49 (0.33-0.72) | 0.0003 |
| Dose: > 1 | -0.1 | 0.74 (0.41-1.33) | 0.31 | -0.1 | 0.85 (0.59-1.22) | 0.38 |
| Cardiovascular |  |  |  |  |  |  |
| All doses | * | 0.43 (0.15-1.20) | 0.11 | 0.0 | 1.18 (0.67-2.09) | 0.57 |
| Dose: 1 | * | 0.50 (0.14-1.77) | 0.28 | * | 1.20 (0.52-2.79) | 0.67 |
| Dose: > 1 | * | 0.33 (0.06-1.99) | 0.23 | * | 1.17 (0.54-2.53) | 0.70 |
| Hospitalization |  |  |  |  |  |  |
| All cause |  |  |  |  |  |  |
| All doses | 0.0 | 0.86 (0.54-1.37) | 0.52 | -0.1 | 0.78 (0.58-1.04) | 0.091 |
| Dose: 1 | * | 0.56 (0.31-1.02) | 0.060 | -0.2 | 0.64 (0.45-0.91) | 0.013 |
| Dose: > 1 | * | 1.80 (0.81-4.02) | 0.15 | 0.0 | 1.25 (0.73-2.15) | 0.42 |
| Cardiovascular |  |  |  |  |  |  |
| All doses | * | 2.00 (0.33-11.97) | 0.45 | * | 0.60 (0.33-1.10) | 0.097 |
| Dose: 1 | * | -- | -- | * | 0.50 (0.24-1.04) | 0.063 |
| Dose: > 1 | * | 1.00 (0.14-7.10) | 1.00 | * | 1.00 (0.32-3.10) | 1.00 |
| Death |  |  |  |  |  |  |
| All doses | * | 1.00 (0.14-7.10) | 1.00 | * | -- | -- |
| Dose: 1 | * | 1.00 (0.14-7.10) | 1.00 | 0.00 | -- | -- |
| Dose: > 1 | 0.00 | -- | -- | * | -- | -- |

“Dose: 1” represents the first dose; “Dose >1” represents all subsequent doses.

CI, confidence interval; CKD, chronic kidney disease; ED, emergency department; HR, hazard ratio; HSR, hypersensitivity reaction.

*Denotes fewer than 10 events contributing. Regulations by the Centers for Medicare & Medicaid Services do not permit display.
