# Supplementary material for: Relative Incidence of Acute Adverse Events with Ferumoxytol Compared to Other Intravenous Iron Compounds: A Matched Cohort Study
Source: PLoS One. 2017 Jan 30;12(1):e0171098. doi: 10.1371/journal.pone.0171098 (PMC5279762; doi:10.1371/journal.pone.0171098)
Supplement: S4 Table — (DOCX) [file pone.0171098.s009.docx]

Table S4. Event risk estimates, for ferumoxytol users versus matched controls, among non-chronic-kidney-disease and non-dialysis-dependent chronic kidney disease patients, derived from the Cox proportional hazards model: outcomes on the day of intravenous iron administration and up to 3 days later

|  | Non-CKD Patients | | | NDD-CKD Patients | | |
| --- | --- | --- | --- | --- | --- | --- |
|  | Incidence Difference, % | HR (95% CI) | *P* | Incidence Difference, % | HR (95% CI) | *P* |
| Anaphylaxis |  |  |  |  |  |  |
| All doses | * | 1.00 (0.43-2.34) | 1.00 | * | 2.00 (0.56-7.09) | 0.28 |
| Dose: 1 | * | 3.00 (0.85-10.63) | 0.089 | * | 0.50 (0.08-2.99) | 0.45 |
| Dose: > 1 | * | 0.33 (0.06-1.99) | 0.23 | * | -- | -- |
| HSR Symptoms |  |  |  |  |  |  |
| All doses | 0.4 | 1.04 (0.94-1.15) | 0.42 | 0.1 | 0.98 (0.89-1.09) | 0.76 |
| Dose: 1 | 0.1 | 0.99 (0.89-1.10) | 0.87 | -0.3 | 0.92 (0.82-1.02) | 0.12 |
| Dose: > 1 | 0.7 | 1.11 (0.96-1.29) | 0.15 | 0.4 | 1.08 (0.93-1.26) | 0.32 |
| Hypotension |  |  |  |  |  |  |
| All doses | 0.0 | 0.91 (0.60-1.39) | 0.67 | 0.0 | 0.93 (0.67-1.29) | 0.65 |
| Dose: 1 | * | 0.77 (0.43-1.38) | 0.38 | 0.0 | 1.04 (0.70-1.55) | 0.84 |
| Dose: > 1 | * | 1.10 (0.62-1.96) | 0.75 | -0.1 | 0.76 (0.46-1.28) | 0.31 |
| ED encounter or hospitalization |  |  |  |  |  |  |
| All cause |  |  |  |  |  |  |
| All doses | -0.4 | 0.74 (0.63-0.87) | 0.0004 | -0.2 | 0.86 (0.77-0.97) | 0.010 |
| Dose: 1 | -0.9 | 0.60 (0.48-0.74) | < 0.0001 | -0.3 | 0.83 (0.72-0.96) | 0.014 |
| Dose: > 1 | 0.1 | 1.00 (0.78-1.29) | 1.0000 | -0.1 | 0.91 (0.76-1.07) | 0.25 |
| Cardiovascular |  |  |  |  |  |  |
| All doses | 0.0 | 0.83 (0.54-1.28) | 0.41 | -0.1 | 0.78 (0.61-0.98) | 0.037 |
| Dose: 1 | -0.1 | 0.61 (0.35-1.05) | 0.077 | -0.1 | 0.84 (0.62-1.15) | 0.27 |
| Dose: > 1 | * | 1.50 (0.71-3.16) | 0.29 | -0.1 | 0.69 (0.48-1.00) | 0.051 |
| ED encounter |  |  |  |  |  |  |
| All cause |  |  |  |  |  |  |
| All doses | -0.3 | 0.68 (0.54-0.85) | 0.0008 | -0.2 | 0.82 (0.70-0.96) | 0.012 |
| Dose: 1 | -0.6 | 0.51 (0.38-0.69) | < 0.0001 | -0.3 | 0.78 (0.63-0.96) | 0.020 |
| Dose: > 1 | 0.0 | 1.00 (0.70-1.43) | 1.0000 | -0.1 | 0.86 (0.68-1.09) | 0.22 |
| Cardiovascular |  |  |  |  |  |  |
| All doses | 0.0 | 0.87 (0.50-1.50) | 0.61 | 0.0 | 0.93 (0.63-1.37) | 0.70 |
| Dose: 1 | * | 0.58 (0.30-1.15) | 0.12 | 0.0 | 1.17 (0.68-2.02) | 0.58 |
| Dose: > 1 | * | 2.00 (0.71-5.62) | 0.19 | -0.1 | 0.73 (0.42-1.28) | 0.27 |
| Hospitalization |  |  |  |  |  |  |
| All cause |  |  |  |  |  |  |
| All doses | -0.2 | 0.78 (0.62-0.98) | 0.035 | -0.1 | 0.88 (0.76-1.02) | 0.087 |
| Dose: 1 | -0.4 | 0.68 (0.50-0.92) | 0.013 | -0.2 | 0.83 (0.68-1.00) | 0.052 |
| Dose: > 1 | 0.0 | 0.94 (0.66-1.33) | 0.72 | 0.0 | 0.96 (0.76-1.21) | 0.73 |
| Cardiovascular |  |  |  |  |  |  |
| All doses | * | 0.64 (0.32-1.26) | 0.20 | -0.1 | 0.68 (0.51-0.92) | 0.011 |
| Dose: 1 | * | 0.50 (0.20-1.22) | 0.13 | -0.1 | 0.69 (0.47-1.00) | 0.048 |
| Dose: > 1 | * | 1.00 (0.32-3.10) | 1.00 | -0.1 | 0.68 (0.43-1.09) | 0.11 |
| Death |  |  |  |  |  |  |
| All doses | * | 1.50 (0.41-5.45) | 0.54 | * | 0.38 (0.13-1.05) | 0.062 |
| Dose: 1 | * | 1.00 (0.25-4.00) | 1.00 | * | 0.67 (0.18-2.42) | 0.54 |
| Dose: > 1 | * | -- | -- | * | 0.20 (0.03-1.24) | 0.084 |

“Dose: 1” represents the first dose; “Dose >1” represents all subsequent doses.

CI, confidence interval; CKD, chronic kidney disease; ED, emergency department; HR, hazard ratio; HSR, hypersensitivity reaction

*Denotes fewer than 10 events contributing. Regulations by the Centers for Medicare & Medicaid Services do not permit display.
