# Supplementary material for: Relative Incidence of Acute Adverse Events with Ferumoxytol Compared to Other Intravenous Iron Compounds: A Matched Cohort Study
Source: PLoS One. 2017 Jan 30;12(1):e0171098. doi: 10.1371/journal.pone.0171098 (PMC5279762; doi:10.1371/journal.pone.0171098)
Supplement: S5 Table — (DOCX) [file pone.0171098.s010.docx]

Table S5. Event risk estimates, for ferumoxytol users versus individually matched users of iron sucrose, ferric gluconate, and iron dextran, among non-chronic-kidney-disease and non-dialysis-dependent chronic kidney disease patients, derived from the Cox proportional hazards model: based on outcomes the day of or the day after iron administration

|  | Non-CKD Patients | | | | | | NDD CKD Patients | | | | | |
| --- | --- | --- | --- | --- | --- | --- | --- | --- | --- | --- | --- | --- |
|  | Ferumoxytol vs Iron Sucrose | | Ferumoxytol vs Ferric Gluconate | | Ferumoxytol vs Iron Dextran | | Ferumoxytol vs Iron Sucrose | | Ferumoxytol vs Ferric Gluconate | | Ferumoxytol vs Iron Dextran | |
|  | HR (95% CI) | *P* | HR (95% CI) | *P* | HR (95% CI) | *P* | HR (95% CI) | *P* | HR (95% CI) | *P* | HR (95% CI) | *P* |
| Anaphylaxis |  |  |  |  |  |  |  |  |  |  |  |  |
| All doses | 1.00 (0.45-2.23) | 1.00 | 0.29 (0.08-1.02) | 0.053 | 0.80 (0.31-2.04) | 0.64 | 2.00 (0.56-7.09) | 0.28 | -- | -- | 0.25 (0.04-1.52) | 0.13 |
| Dose: 1 | 1.00 (0.32-3.10) | 1.00 | 0.29 (0.08-1.02) | 0.053 | 0.75 (0.26-2.18) | 0.60 | 1.00 (0.14-7.10) | 1.00 | -- | -- | 0.25 (0.04-1.52) | 0.13 |
| Dose: > 1 | 1.00 (0.32-3.10) | 1.00 | -- | -- | 1.00 (0.14-7.10) | 1.00 | 3.00 (0.50-17.95) | 0.23 | -- | -- | -- | -- |
| HSR symptoms |  |  |  |  |  |  |  |  |  |  |  |  |
| All doses | 1.17 (1.05-1.31) | 0.0045 | 0.76 (0.67-0.86) | < 0.0001 | 0.82 (0.73-0.91) | 0.0002 | 1.09 (0.97-1.22) | 0.14 | 0.54 (0.45-0.65) | < 0.0001 | 0.46 (0.40-0.53) | < 0.0001 |
| Dose: 1 | 1.18 (1.06-1.33) | 0.0034 | 0.71 (0.63-0.80) | < 0.0001 | 0.84 (0.76-0.93) | 0.0012 | 1.08 (0.96-1.23) | 0.20 | 0.61 (0.51-0.73) | < 0.0001 | 0.51 (0.44-0.59) | < 0.0001 |
| Dose: > 1 | 1.16 (1.00-1.34) | 0.056 | 0.83 (0.70-1.00) | 0.054 | 0.76 (0.63-0.93) | 0.0080 | 1.10 (0.93-1.29) | 0.26 | 0.46 (0.34-0.63) | < 0.0001 | 0.37 (0.29-0.47) | < 0.0001 |
| Hypotension |  |  |  |  |  |  |  |  |  |  |  |  |
| All doses | 1.00 (0.61-1.65) | 1.00 | 0.32 (0.20-0.51) | < 0.0001 | 2.14 (1.09-4.21) | 0.027 | 1.32 (0.84-2.07) | 0.23 | 0.30 (0.16-0.56) | 0.0002 | 1.25 (0.63-2.49) | 0.53 |
| Dose: 1 | 0.80 (0.41-1.55) | 0.51 | 0.18 (0.09-0.36) | < 0.0001 | 1.60 (0.71-3.60) | 0.26 | 1.15 (0.68-1.95) | 0.59 | 0.33 (0.17-0.66) | 0.0015 | 1.17 (0.54-2.53) | 0.70 |
| Dose: > 1 | 1.25 (0.57-2.75) | 0.58 | 1.00 (0.50-2.00) | 1.00 | 3.50 (0.98-12.48) | 0.053 | 1.67 (0.78-3.58) | 0.19 | 0.25 (0.09-0.71) | 0.0092 | 1.50 (0.41-5.45) | 0.54 |
| ED encounter or hospitalization |  |  |  |  |  |  |  |  |  |  |  |  |
| All-cause |  |  |  |  |  |  |  |  |  |  |  |  |
| All doses | 0.63 (0.52-0.77) | < 0.0001 | 0.25 (0.20-0.31) | < 0.0001 | 0.72 (0.57-0.91) | 0.0064 | 0.90 (0.78-1.04) | 0.14 | 0.26 (0.20-0.33) | < 0.0001 | 0.72 (0.57-0.91) | 0.0054 |
| Dose: 1 | 0.45 (0.34-0.60) | < 0.0001 | 0.16 (0.11-0.22) | < 0.0001 | 0.67 (0.50-0.89) | 0.0059 | 0.84 (0.69-1.02) | 0.079 | 0.21 (0.16-0.29) | < 0.0001 | 0.69 (0.52-0.92) | 0.011 |
| Dose: > 1 | 0.98 (0.73-1.31) | 0.88 | 0.61 (0.44-0.85) | 0.0034 | 0.85 (0.57-1.26) | 0.43 | 0.98 (0.78-1.21) | 0.82 | 0.43 (0.27-0.69) | 0.0004 | 0.79 (0.53-1.17) | 0.23 |
| Cardiovascular |  |  |  |  |  |  |  |  |  |  |  |  |
| All doses | 0.86 (0.50-1.48) | 0.58 | 0.19 (0.11-0.32) | < 0.0001 | 0.73 (0.38-1.40) | 0.34 | 0.76 (0.56-1.04) | 0.088 | 0.19 (0.11-0.34) | < 0.0001 | 0.58 (0.34-1.00) | 0.049 |
| Dose: 1 | 0.56 (0.25-1.24) | 0.15 | 0.09 (0.04-0.21) | < 0.0001 | 0.45 (0.20-1.01) | 0.053 | 0.91 (0.60-1.39) | 0.67 | 0.11 (0.05-0.25) | < 0.0001 | 0.62 (0.32-1.17) | 0.14 |
| Dose: > 1 | 1.40 (0.61-3.19) | 0.42 | 0.78 (0.38-1.57) | 0.48 | -- | -- | 0.61 (0.38-0.99) | 0.044 | 0.71 90.31-1.63) | 0.42 | 0.50 (0.18-1.40) | 0.19 |
| ED encounter |  |  |  |  |  |  |  |  |  |  |  |  |
| All-cause |  |  |  |  |  |  |  |  |  |  |  |  |
| All doses | 0.54 (0.42-0.70) | < 0.0001 | 0.40 (0.30-0.54) | < 0.0001 | 0.71 (0.53-0.96) | 0.027 | 0.72 (0.59-0.87) | 0.0008 | 0.67 (0.49-0.93) | 0.017 | 1.14 (0.83-1.55) | 0.43 |
| Dose: 1 | 0.31 (0.21-0.47) | < 0.0001 | 0.23 (0.15-0.36) | < 0.0001 | 0.58 (0.39-0.86) | 0.0073 | 0.65 (0.50-0.85) | 0.0016 | 0.56 (0.37-0.83) | 0.0042 | 1.08 (0.73-1.59) | 0.70 |
| Dose: > 1 | 1.07 (0.74-1.56) | 0.71 | 0.95 (0.60-1.51) | 0.84 | 1.00 (0.61-1.63) | 1.00 | 0.80 (0.60-1.08) | 0.14 | 1.00 (0.58-1.72) | 1.00 | 1.25 (0.73-2.15) | 0.42 |
| Cardiovascular |  |  |  |  |  |  |  |  |  |  |  |  |
| All doses | 0.89 (0.45-1.75) | 0.73 | 0.44 (0.22-0.86) | 0.016 | 1.00 (0.42-2.40) | 1.00 | 0.65 (0.42-1.02) | 0.059 | 0.50 (0.22-1.14) | 0.10 | 1.00 (0.50-2.00) | 1.00 |
| Dose: 1 | 0.60 (0.21-1.70) | 0.34 | 0.25 (0.09-0.71) | 0.0092 | 0.60 (0.21-1.70) | 0.34 | 0.77 (0.43-1.38) | 0.38 | 0.25 (0.07-0.90) | 0.034 | 1.40 (0.61-3.19) | 0.42 |
| Dose: > 1 | 1.25 (0.49-3.19) | 0.64 | 1.00 (0.38-2.66) | 1.0000 | -- | -- | 0.54 (0.27-1.06) | 0.074 | 1.50 (0.41-5.45) | 0.54 | 0.33 (0.06-1.99) | 0.29 |
| Hospitalization |  |  |  |  |  |  |  |  |  |  |  |  |
| All-cause |  |  |  |  |  |  |  |  |  |  |  |  |
| All doses | 0.72 (0.53-0.99) | 0.045 | 0.16 (0.11-0.23) | < 0.0001 | 0.73 (0.51-1.04) | 0.082 | 1.09 (0.88-1.35) | 0.44 | 0.14 (0.09-0.21) | < 0.0001 | 0.38 (0.26-0.56) | < 0.0001 |
| Dose: 1 | 0.66 (0.43-0.99) | 0.047 | 0.12 (0.07-0.18) | < 0.0001 | 0.76 (0.50-1.16) | 0.21 | 1.04 (0.79-1.38) | 0.77 | 0.13 (0.08-0.21) | < 0.0001 | 0.38 (0.23-0.61) | < 0.0001 |
| Dose: > 1 | 0.83 (0.51-1.36) | 0.46 | 0.38 (0.22-0.64) | 0.0003 | 0.67 (0.35-1.27) | 0.22 | 1.15 (0.83-1.60) | 0.40 | 0.17 (0.07-0.43) | 0.0002 | 0.39 (0.20-0.76) | 0.0061 |
| Cardiovascular |  |  |  |  |  |  |  |  |  |  |  |  |
| All doses | 1.00 (0.42-2.40) | 1.00 | 0.13 (0.06-0.27) | < 0.0001 | 0.67 (0.27-1.66) | 0.38 | 0.82 (0.53-1.27) | 0.37 | 0.11 (0.05-0.26) | < 0.0001 | 0.23 (0.08-0.66) | 0.0060 |
| Dose: 1 | 0.50 (0.14-1.77) | 0.28 | 0.05 (0.01-0.18) | < 0.0001 | 0.33 (0.09-1.18) | 0.089 | 1.00 (0.55-1.81) | 1.00 | 0.08 (0.03-0.23) | < 0.0001 | 0.10 (0.02-0.65) | 0.016 |
| Dose: > 1 | 3.00 (0.50-17.95) | 0.23 | 0.80 (0.31-2.04) | 0.64 | -- | -- | 0.64 (0.32-1.26) | 0.20 | 0.40 (0.11-1.41) | 0.15 | 0.67 (0.18-2.42) | 0.54 |
| Death |  |  |  |  |  |  |  |  |  |  |  |  |
| All doses | 2.00 (0.33-11.97) | 0.45 | 0.33 (0.06-1.99) | 0.23 | -- | -- | 0.50 (0.08-2.99) | 0.45 | -- | -- | -- | -- |
| Dose: 1 | 2.00 (0.33-11.97) | 0.45 | 0.33 (0.06-1.99) | 0.23 | -- | -- | -- | -- | -- | -- | -- | -- |
| Dose: > 1 | -- | -- | -- | -- | -- | -- | 1.00 (0.14-7.10) | 1.00 | -- | -- | -- | -- |

“Dose: 1” represents the first dose; “Dose >1” represents all subsequent doses.

CI, confidence interval; CKD, chronic kidney disease; ED, emergency department; HR, hazard ratio; HSR, hypersensitivity reaction.
